# Ileo-colic intussusception: a rare cause of intestinal obstruction in adults

**DOI:** 10.1093/jscr/rjaf723

**Published:** 2025-09-09

**Authors:** Mintimer Negametzyanov, Christopher R Smith, Efthymios Ypsilantis

**Affiliations:** Department of Colorectal Surgery, Princess Royal University Hospital, Kings College Hospital NHS Foundation Trust, Farnborough Common, Orpington, Kent, BR6 8ND, United Kingdom; Department of Colorectal Surgery, Princess Royal University Hospital, Kings College Hospital NHS Foundation Trust, Farnborough Common, Orpington, Kent, BR6 8ND, United Kingdom; Department of Colorectal Surgery, Princess Royal University Hospital, Kings College Hospital NHS Foundation Trust, Farnborough Common, Orpington, Kent, BR6 8ND, United Kingdom

**Keywords:** intussusception, intestinal obstruction, colectomy

## Abstract

Adult intussusception is a rare cause of intestinal obstruction characterized by the telescoping of one segment of the intestine into another. Unlike in children, adult intussusception accounts for a small proportion of intestinal obstructions and is usually associated with an identifiable cause, such as malignancy or polyps. We present a case of ileo-colic intussusception in an elderly female, confirmed on abdominal computed tomography (CT) imaging, and successfully treated with emergency laparotomy and segmental bowel resection. Histology identified a benign lipomatous polyp as the lead point for the intussusception. This case demonstrates that adult bowel intussusception presents with non-specific symptoms, and early detection through abdominal CT imaging is crucial. Surgeons should be aware of the potential association with underlying malignancy, particularly in elderly patients, and therefore segmental resection should follow strict oncological principles.

## Introduction

Intussusception is a rare cause of intestinal obstruction in adults. It is defined as the telescoping an intestinal segment into the lumen of an adjacent segment. Unlike in children, adult intussusception is associated with an identifiable cause, such as malignancy, polyps, or inflammatory lesions, in 90% of cases [1]. The pathologic lesion acts as a ‘lead point’, a focal area of traction drawing one intestinal segment into another. Presentation can be non-specific but is generally with symptoms of small or large bowel obstruction. Most cases (70%–90%) require definitive treatment, most commonly with surgical resection [2].

## Case report

A 79-year-old female presented to the Emergency Department with bleeding per rectum. She also reported 1 week of dull, achy, upper abdominal pain associated with loss of appetite, nausea, and vomiting. She had no history of unintentional weight loss. She has a history of hypercholesterolemia and osteoarthritis, and no history of abdominal surgery. She is an ex-smoker and consumes alcohol occasionally. She has no significant family history.

On admission, she was apyrexial, normotensive, and not tachycardic (84 bpm). On physical examination, her abdomen was soft but distended. Digital rectal examination did not reveal any anorectal masses. Blood tests identified raised C-reactive protein (31 mg/ml) and a stage 1 acute kidney injury with eGFR 49 ml/min/1.73 m^2^. Full blood count, electrolytes, and liver function tests were unremarkable.

**Figure 1:**
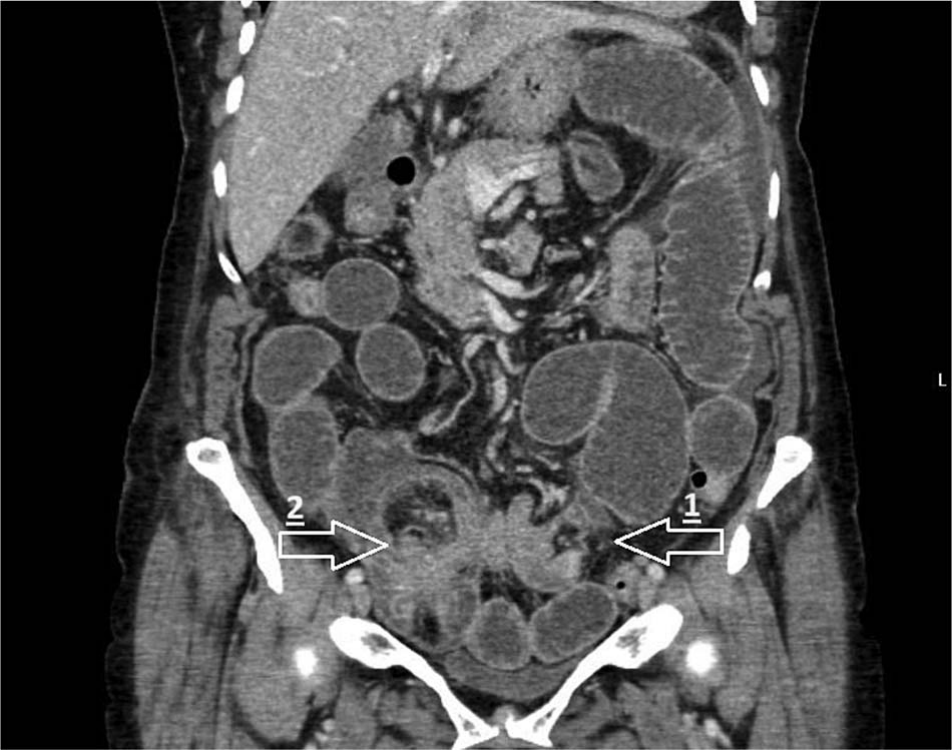
CT coronal image demonstrating small bowel obstruction secondary to ileo-colic intussusception with large segment of small bowel invaginating into caecum. Arrows mark the following: 1—transition point of bowel obstruction, 2—ileo-caecal intussusception ‘lead point’.

Computed tomography (CT) of the abdomen and pelvis showed high-grade small bowel obstruction secondary to ileo-caecal intussusception with a large segment of the small bowel and its mesentery invaginating into the caecum (Fig. 1).

**Figure 2:**
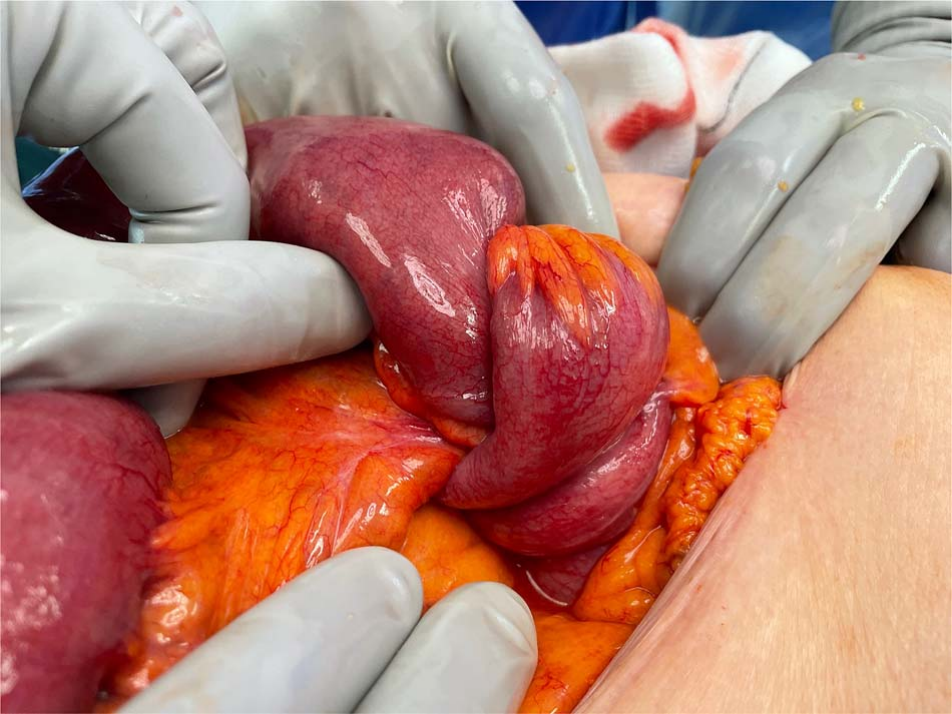
Intra-operative photograph showing ileo-colic intussusception—the small bowel and its mesentery is seen invaginating into the caecum.

Given these findings, the patient went to theatre for an emergency laparotomy. Intra-operative findings were of ileo-caecal intussusception with dilation of the proximal small bowel to the point of intussusception (Fig. 2). There was no evidence of bowel ischaemia or perforation. Palpation of the mesentery revealed multiple enlarged lymph nodes, which were felt to be reactive in nature. An ileo-colic resection was performed to resect the area of intussuscepted bowel without attempted reduction. This was followed by a side-to-side stapled ileo-colic anastomosis.

The patient developed hypomagnesaemia and hypophosphataemia postoperatively, requiring intravenous replacement, but her postoperative course was otherwise uneventful. Her renal function normalised, and she was discharged on postoperative day 6.

The histopathology report confirmed ileo-colic intussusception and identified a 35 mm polyp as the lead point. Microscopic examination of the polyp revealed submucosal mature adipocytes consistent with a conventional lipoma, with no evidence of dysplasia or malignancy. The intussuscepted segment of bowel measured 95 mm in length and had microscopic findings showing focal inflammation and mucosal ulceration, consistent with an ischaemic type of mucosal injury.

The patient was followed up in the outpatient clinic 10-weeks postoperatively, had recovered well and had no further gastro-intestinal symptoms. A colonoscopy was performed routinely which showed a tight diverticular sigmoid. A paediatric colonoscope was therefore used to complete the procedure, which identified two small hyperplastic colonic polyps (<5 mm) but otherwise normal appearances to the ileo-colic anastomosis. The colonic polyps were removed endoscopically. She was seen in outpatient setting for further surveillance and subsequently discharged from the hospital follow-up.

## Discussion

First described by Barbette [3], and subsequently in greater detail by John Hunter [4] as ‘introssusception’, intussusception accounts for 1%–5% of all cases of intestinal obstruction in adults [1]. We present a case of ileo-colic intussusception, secondary to a benign lipomatous polyp in an elderly female presenting with non-specific gastrointestinal symptoms. The acute diagnosis was made on CT scan, though the underlying cause for the intussusception was unclear, as is often the case. The most common underlying cause in this age group is neoplastic, accounting for two-thirds of cases [5].

In our case, the patient was managed with emergency laparotomy and surgical resection of the affected bowel segment. There is a consensus that in such cases, particularly in patients above the age of 60 years, surgical resection should be performed following strict oncological principles, without prior manipulation or reduction of the intussuscepted segment, due to an increased risk of tumour dissemination, perforation, seeding, and anastomotic complications [6, 7].

A vast array of benign and malignant causes of intussusception have been described in the literature and can be further classified into intra-luminal or extra-luminal lesions. In our case, histological analysis of the resected bowel segment identified the likely ‘lead point’ for her intussusception as an intestinal lipoma, though the clinician must always consider the possibility of underlying primary bowel malignancy (adenocarcinoma). Other notable causes described in the wider literature include inflammatory lesions, Meckel’s diverticulum, adenomatous polyps, lymphoma, and metastases [8].

## Conclusion

Adult bowel intussusception is a rare but noteworthy cause of intestinal obstruction in adults. Pre-operative diagnosis can be delayed or missed due to non-specific clinical presentation. Prompt abdominal CT imaging is vital for early diagnosis. Surgeons must be mindful of the frequent association between ileo-colic intussusception and underlying malignant lesions. Surgical intervention is therefore often necessary, in the form of segmental resection following strict oncological principles, without attempted manipulation or reduction of the affected bowel segment.
